# History, Pathogenesis, and Management of Familial Gastric Cancer: Original Study of John XXIII's Family

**DOI:** 10.1155/2013/385132

**Published:** 2012-12-26

**Authors:** Giovanni Corso, Fabrizio Roncalli, Daniele Marrelli, Fátima Carneiro, Franco Roviello

**Affiliations:** ^1^Department of Human Pathology and Oncology, Section of General Surgery and Surgical Oncology and Translational Research Laboratory, University of Siena, Viale Bracci, 53100 Siena, Italy; ^2^Azienda Sanitaria Locale, Bergamo Province, Via alla Guardina 24039, Sotto il Monte Giovanni XXIII, Italy; ^3^Medical Faculty of the University of Porto, st. John Hospital Center and Institute of Molecular Pathology and Immunology of the University of Porto (IPATIMUP), rua dr. Roberto Frias 4200-465 Porto, Portugal

## Abstract

*Background*. Hereditary diffuse gastric cancer is associated with the E-cadherin germline mutations, but genetic determinants have not been identified for familial intestinal gastric carcinoma. The guidelines for hereditary diffuse gastric cancer are clearly established; however, there are no defined recommendations for the management of familial intestinal gastric carcinoma. *Methods*. In this study we describe Pope John XXIII's pedigree that harboured gastric cancer as well as six other family members. Family history was analysed according to the International Gastric Cancer Linkage Consortium criteria, and gastric tumours were classified in accord with the last Japanese guidelines. *Results*. Seven out of 109 members in this pedigree harboured gastric cancer, affecting two consecutive generations. John XXIII's clinical tumour (cTN) was classified as cT4bN3a (IV stage). In two other cases, gastric carcinomas were classified as intestinal histotype and staged as pT1bN0 and pT2N2, respectively. *Conclusions*. Pope John XXIII's family presents a strong aggregation for gastric cancer affecting almost seven members; it spreads through two consecutive generations. In absence of defined genetic causes and considering the increased risk of gastric cancer's development in these families, as well as the high mortality rates and advanced stages, we propose an intensive surveillance protocol for asymptomatic members.

## 1. Introduction

About 80–90% of gastric carcinomas develop in a sporadic setting, the remaining 10% to 20% show familial cluster, and approximately only 1–3% have a clear inherited genetic conditioning [1–4]. In literature there are many reports of familial gastric cancer (FGC) with no evidence of cancer in other organs, encompassing both hereditary forms and GC clustering in families without determinant genetic susceptibility for the disease [1, 5–7].

E-cadherin gene (*CDH1*) mutations were identified as the causal event underlying the hereditary diffuse gastric cancer (HDGC) syndrome [8]. The guidelines for the management of the HDGC familial members were established by the International Gastric Cancer Linkage Consortium (IGCLC) in 1999 [2] and updated in 2010 [3]. *TP53* or mismatch repair gene (MMR) germline mutations account, respectively, for Li-Fraumeni and Lynch syndromes and, in these settings, gastric carcinoma, may develop in association with neoplastic diseases in other organs [4, 9–11].

Though the guidelines for families' management with HDGC are clearly established [3], there are no specific recommendations for families' management with other FGC's types, namely, familial intestinal gastric cancer (FIGC).

Herein we report Pope John XXIII's pedigree, displaying a clear excess of family members harbouring GC, with intestinal histotype and without cancer evidence in other organs. Furthermore, we suggest a surveillance and management for living kindred, to minimize the cancer risk in this family.

## 2. Methods

### 2.1. Familial History

Data on the family history were collected by direct interview of living members and consulting historical documents, obtained from John XXIII's personal archives. Briefly, the closest relatives were asked to report the total number of relatives in John XXIII's family (Pope John identified the proband), their ages, and their living status and the members harbouring gastric tumour, age at onset of disease, death date, and cancers in other organs. Familial aggregation was investigated with particular reference to the IGCLC criteria [2, 3]. In particular, for FIGC definition, we considered these criteria: (a) at least three relatives should have intestinal GC and one of them should be a first degree relative of the other two; (b) at least two successive generations should be affected; (c) in one of the relatives, GC should be diagnosed before the age of 50 [2].

### 2.2. Clinicopathological Data

**Figure 1:**
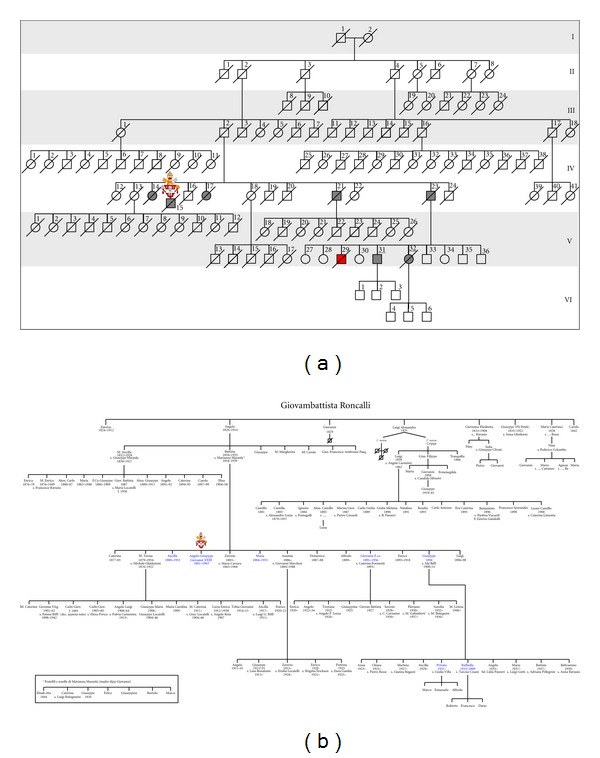
(a) Schematic pedigree of John XXIII's family with seven cases affected by primary gastric carcinoma (generations IV and V). Clinicopathological information were available for cases marked with underline numbers; (b) Roncalli's original pedigree, firstly described in 1968. The bold characters indicated members affected by primary gastric tumours; the proband was indicated with the papal shield.

Clinicopathological information were available for three members affected by primary gastric carcinoma, as illustrated in Figure 1(a) (cases IV-15, V-31, and V-32). For these cases, information about diagnosis, surgical procedure, histopathological examination, and survival were available. Regarding the proband (John XXIII), clinicopathological information were collected by consulting historical documents obtained from John XXIII's museum (Ca' Maitino museum, Sotto il Monte Giovanni XXIII, Bergamo, Italy).

Informed consent was obtained from all subjects included in this study and approved by hospital's ethics committee.

## 3. Results

### 3.1. Pedigree Analysis

Figures 1(a) and 1(b) represent the complete Roncalli family. In total, 109 members were identified, belonging to six generations. There were 66 males (60.6%) and 43 females (39.4%). Seven members were affected by gastric carcinoma; two out of six consecutive generations (IV and V) were involved. One single case of sporadic bladder cancer was identified (V-29). The GC overall frequency in this family was rather high (7/109), also considering that only two generations (IV and V) were affected. The generation IV showed the highest frequency for GC aggregation (5/41), decreasing to two GC cases in the next generation (V). So far, the last explored generation (VI) is cancer-free. Among GC patients there were four males (57.1%) and three females (42.9%); the overall mean age at onset was 75.8 years, 78.2 for males and 72.6 years for females, respectively. The youngest and the oldest ages at onset were 65 and 87 years, respectively. GC mortality rate in this family was rather high, with six of seven patients having died from causes related to tumour metastasis.

### 3.2. The Clinical History of John XXIII (Case IV-15)

**Figure 2:**
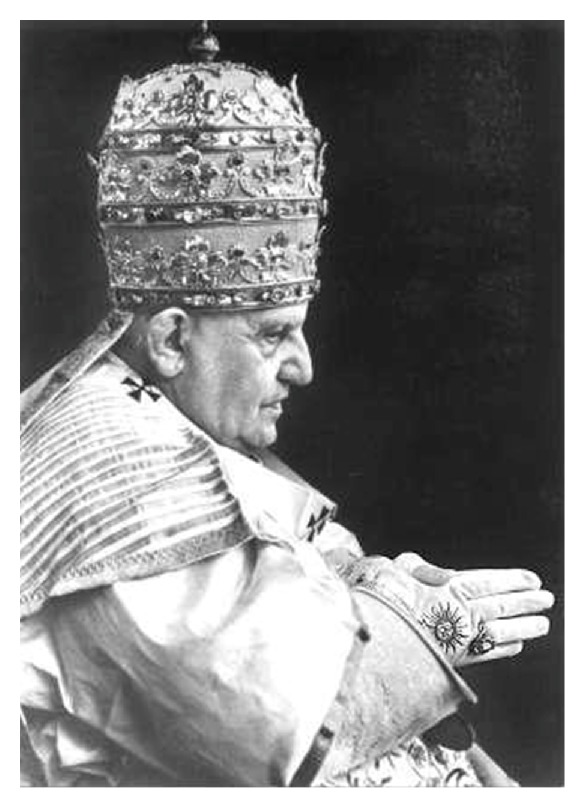
Coronation day, November 1958. Pope John XXIII with pontiff vestments wearing the papal tiara and “fanon” that defines the supreme authority as Roman Pontiff of the Catholic Church.

Pope John XXIII was born in Angelo Giuseppe Roncalli at Sotto il Monte (Bergamo) in Italy, on 25 November 1881. He was the fourth in a family of 13 children (Figures 1(a) and 1(b)). On August 1904 he was ordained as priest in Rome, and in 1925 Pope Pius XI named him apostolic visitator in Bulgaria, raising him to the episcopate. In 1953 he was appointed cardinal of Venice, finally at Pope Pius XII's death, Angelo Roncalli was elected Roman Pontiff on 28, October 1958, taking the name John XXIII (Figure 2). During that time, in October 1962, John XXIII convoked the Ecumenical Vatican Council II.

The clinical history began in September 1962. Firstly, he complained dyspepsia, sporadic episodes of vomiting, and weight loss (about 5 kg). X-ray examination revealed a distal gastric tumour narrowing the antrum and the angulus with pylorus substenosis and wall ulceration. Main symptoms referred by the Pope are described in detail in Table 1.

The papal physician, namely *archiatre*, convoked three eminent Italian surgeons, that visited the Pope in the papal apartments and described a palpable mass in right hypochondrium with abdominal ascites; considering the aged patient, the obesity, and other comorbidities, collegially they defined the tumour as inoperable deciding for a conservative/palliative approach. In particular, a surgeon assessed that the mortality risk for an extended gastrectomy was earnest high and whenever a radical intent was possible, the long survival's probability was very low. The conservative treatments were routinely blood and plasma transfusions, gastric mucosa extract (Opogastrina), cyclophosphamide (Endoxan), antianemic agent (Hepavis), and procoagulant drugs.

Considering these clinical reports, we could asses that the gastric tumour staging was cT4bN2 (IV stage) with intestinal histotype, because of later age at onset and slow tumour progression. However, histopathological confirmation was not available.

Pope John XXIII died in Vatican City in the evening of June 3, 1963, from peritonitis due to gastric carcinoma perforation. John XXIII's body was treated with chemical agents (fomaldheyde) to prevent the postmortem corruption; about 5 liters of abdominal ascites were drained.

### 3.3. Case V-31

Male, 79 years, was admitted at Bergamo's hospital (Italy) after incidental discovery at endoscopy of a suspicious gastric lesion; the histopathological examination of biopsies diagnosed an adenocarcinoma. There was no metastasis' evidence in other organs. The patient suffered from colon diverticular disease, abdominal aortic aneurism (treated with endovascular stent), hypertension, and prostatic hypertrophy. The patient was submitted to total gastrectomy and the pathological examination described gastric adenocarcinoma (intestinal histotype), G2 grading, with invasion of the submucosa, pT1bN0 staging. The patient is alive and well, with no evidence of local relapses or distant metastases.

### 3.4. Case V-32

Female, 74 years, referred vomits, nausea, diarrhoea, and body weight loss (about 15 kg). At endoscopy an infiltrative tumour was identified, causing stenosis and extending to the duodenum. The patient was submitted to subtotal gastrectomy with gastrojeiunostomy (Roux reconstruction). Due to a postoperative complication, the patient was reoperated and a total gastrectomy was performed. The pathological examination revealed gastric adenocarcinoma (intestinal histotype), G3 grading, with venous and perineural invasion. The tumour invaded the muscle layer and nodal metastases were identified in 7 out of 23 perigastric lymph nodes (pTNM stage was pT2N3a). The patient was submitted to adjuvant chemotherapy and died two years after surgery, with massive peritoneal carcinomatosis and hepatic metastases.

## 4. Management and Endoscopic Surveillance

### 4.1. Clinical Setting

A familial history as the one herein described raises several relevant issues regarding management and clinical surveillance of the asymptomatic familial members. This family fulfils the criteria for FIGC, according to the IGCLC definitions [2]. As such, this family does not qualify for the screening of E-cadherin gene (*CDH1*) germline mutations which should be offered to families with HDGC [3] and early onset GC (diffuse histotype) [12]. Moreover, the pedigree analysis excluded the possibility of Li-Fraumeni or Lynch syndromes, such as *TP53* or MMR genes' screening for a germline mutation that was not performed [4]. However, the familial members are at increased risk of GC development and management's strategy and clinical surveillance is mandatory in this family in order to reduce morbidity and mortality.

### 4.2. Endoscopic Surveillance

**Figure 3:**
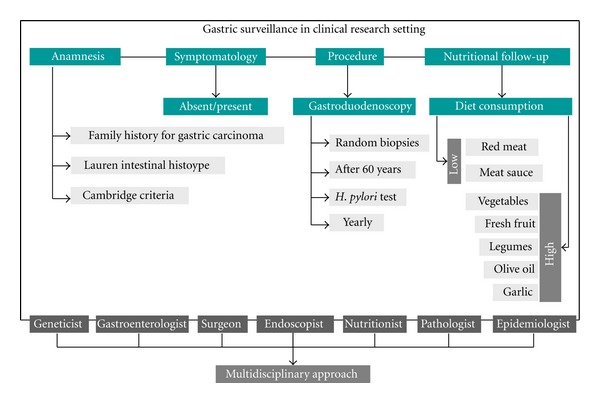
The proposed flow chart is suggested for the gastric surveillance in asymptomatic members recorded in this pedigree and in cases with familial intestinal gastric cancer. Some indications, such as the age for the first gastroendoscopy, are specific for this pedigree.

Based on the guidelines recently proposed by Kluijt and collaborators [13], we developed a protocol surveillance for asymptomatic members in this novel pedigree (Figure 3). Specifically, these guidelines recommended gastroduodenoscopy at age of 40 years (or at an age 5 years younger than youngest diagnosis in a family) with *Helicobacter pylori* testing and eradication. Attention should be given also to diet habits, namely, in GC high incidence areas and in cases with familial aggregation, based on the available evidence that indicates that specific foods, such as high consumption of grilled red meat and meat sauce, increase the risk of familial GC development [14].

Accordingly, for the family herein reported, we recommend a multidisciplinary approach with genetic counselling (Figure 3). Taking into consideration the age at onset and gender of affected kindred, as well as the GC high frequency, we suggest a periodic endoscopic surveillance, beginning at 60 years, even in the absence of symptoms. The optimal endoscopic interval is an important parameter to define. A Japanese study analysed the association between the interval of upper gastrointestinal endoscopies and the GC stage at diagnosis in patients from a GC high prevalence and in families with GC clustering [15]. These authors verified that the risk was not increased in patients in the 2- or 3-year interval group, whereas it was increased in the 4- or 5-year interval groups. In familial cases, the authors observed that in patients with a GC familial history, the risk of a GC higher stage at diagnosis was greater in patients who had a 3-year interval between endoscopies than in those with a 1-year interval and probably higher than in those with a 2-year interval. Similarly, these authors confirmed that the age of 60 years for the first endoscopy represents a valid age cut-off, particularly in families clustering for GC with abundance of intestinal histotype [16]. Other studies confirmed the utility of yearly endoscopy as the optimal interval also in other Eastern populations [15].

Thus, we suggested for this family an endoscopic yearly periodic interval. Moreover, medical examination and detailed interviews should be performed before the endoscopic procedures. Endoscopy should be performed using a white light high definition endoscope in a dedicated session with at least 30 min allocated to allow a careful inspection of the mucosa on inflation and deflation, and to allow time for multiple biopsies to be taken. Use of mucolytics such as acetylcysteine may be helpful to obtain good views [3]. Further, chromoendoscopy constitutes also an option [17]. Besides random or geographically targeted biopsies, all suspicious lesions should be biopsied [18].

## 5. Discussion

In 1964, Jones cited in literature a pedigree with FGC aggregation [7], corresponding to two families collected by Paulsen in 1924; in one of these families, the father, the mother and six children harboured gastric carcinoma; in the other family, the mother, and five children were affected. In 1938, Napoleon Bonaparte's family was reported [5], in which several members were affected by assured (Napoleon and his father) or suspicious GC (the grandfather, one brother, and four sisters). In 1958, Graham and Lilienfeld [6] performed genetic studies and statistical analysis of cancer developing in mono- and dizygotic twins; they found that in some specific sites, such as the stomach, if GC develops in monozygotic twin, there is an increased risk for the GC development in the other twin. In 1964, Jones identified a Maori family with a high frequency of GC; in a pedigree with 98 members, 28 were affected by primary gastric carcinoma and, within a period of 30 years, over 25 subjects died from this disease [7]. GC with familial cluster, in absence of other tumours, led to the search for genetic or environmental risk factors that are associated with familial GC development's risk. In 1998, Guilford and collaborators identified, for the first time, that E-cadherin gene (*CDH1*) germline mutations constitute the genetic cause of HDGC [8]. It is now known that HDGC penetrance is about >80% [3].

Several studies showed that a familial history of GC is a risk factor for the development of the disease [19–26]. Having a first-degree relative with GC is a risk factor for GC development with odds ratio (OR) varying 2 to 10 according to the geographic region and ethnicity [27]. A large study from Turkey conferred an OR 10.1 for GC patients' siblings; nevertheless the results were not adjusted for environmental factors [28]. However, when this adjustment for environmental factors was done, it did not alter the risk. Interestingly, the Lauren GC intestinal histotype was more strongly associated with the GC familial history than the diffuse histotype [18, 23, 29].

A positive family history is considered a strong risk factor for GC development. Except for HDGC, the molecular basis for the familial aggregation is largely unknown [27].

It is believed that this GC familial cluster is due to a genetic susceptibility, shared environmental or lifestyle factors, or a combination of these in different populations. Current data shows a GC increased risk for relatives of GC patients and, in the other hand, an increased prevalence of *Helicobacter pylori* infection and premalignant lesions. There are no studies aimed to assess if the premalignant lesions of GC patient's relatives progress more rapidly through the carcinogenic cascade to GC than premalignant lesions in matched controls of general population [30]. However, so far it was not possible to identify a specific genetic cause for FIGC [1, 29]. New families with FIGC constitute nature's models that, in the future, may lead to the identification of genetic cause(s) and determinant environmental risk factors for this syndrome. Currently, it is recognized that patients at increased risk for GC due to ethnic background or familial history may benefit from surveillance [31]. Accordingly, GC familial history should be taken into account in the followup of precancerous conditions and lesions of the stomach. The Dutch working group on HGC has formulated guidelines for various aspects of medical management for families and individuals at high risk of GC developing, including criteria for referral, classification, diagnostics, and periodic gastric surveillance [13]. We took into consideration all these recommendations for the multidisciplinary protocol's design and for the asymptomatic members' surveillance of the family herein reported.

Detailed pedigrees, constructed with at least three generations, can provide important information for this purpose.

**Figure 4:**
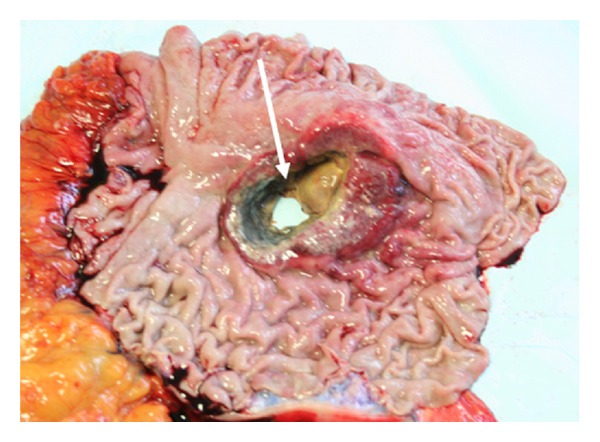
Representative sample of perforated gastric tumour (personal archive); arrow indicates the depth perforation.

In the present study we described the GC history of Pope John XXIII and his family that was firstly recorded in 1968 (Figure 1) (Capovilla, Letters to family (1901–1962)). In this pedigree seven stomach cancer's cases in two consecutive generations were identified. By clinical history's evaluation and historical documents' exploration, it was concluded that Pope John XXIII died from a perforated GC staged at least as cT4bN3a. Perforation is a rare gastric carcinoma's complication, occurring in less than 1% of GC cases (Figure 4). In most cases, the tumour invades the serosa and displays metastatic lymph nodes in second level. The process of gastric wall perforation is sustained by infectious and ischemic factors due to the tumour neovascularisation which result in the shedding of the neoplastic tissue [32]. In this family we observed that GC appeared only in fourth and in fifth generations (XIX-XX centuries), with the highest frequency in the fourth generation. Most probably, along a time frame of about one century, this family was exposed to the same risk factors, such as environmental agents and diet habits. The putative role of genetic susceptibility and/or epigenetic changes can not be excluded.

## 6. Conclusions

Within familial cases, FIGC is a well recognized disease though its pathogenesis has not been fully elucidated yet. The identification of families fulfilling the criteria for FIGC requires a careful surveillance for asymptomatic members in these families. In this study we report Pope John XXIII's family, a historical family with a GC high frequency, displaying the features of intestinal carcinoma. In absence of elected genetic screening, such as searching for E-cadherin germline mutations, we proposed a pedigree-specific surveillance in asymptomatic kindred in accordance with recent guidelines. Instead, in truncating *CDH1* germline mutation carriers, prophylactic total gastrectomy represents the only life saving treatment.

## Figures and Tables

**Table 1:** Clinicopathological approach to John XXIII's gastric illness (Vatican City 1962-1963). As shown in this table, we considered four clinical phases.

Features	September-October 1962 Phase 1	November-December 1962 Phase 2	January–April 1963 Phase 3	May-June 1963 Phase 4
Clinical symptoms/signs	Dyspepsia, vomits, weight loss (5 kg in 4 years)	Epigastric pain, palpable mass in right hypocondrium, anemia, severe postprandial pain, nocturnal epigastric pain, insomnia, acute haemorrhage, severe anemia	Persistent epigastric pain, anorexia	Chronic epigastric pain with frequent exacerbations, multiple episodes of vomits and bleeding, melenas, strong widespread pain, anemia

Examinations/procedures	X-ray: tumour narrowing the antral region of the stomach, pyloric stenosis Ulceration	Blood and plasma transfusions, B12 vitamin, batroxobine cyclophosphamide, bicarbonate	Clinical followup	Ascites (5 litres), blood transfusion

Diagnosis/pathogenesis/evolutions	Family history	Advanced gastric cancer, cT4bN2 (IV stage), Intestinal histotype?	Unresectable gastric carcinoma	Tumour perforation, peritonitis, fever, coma, death
